# A case of anterior mediastinal mature teratoma with severe inflammatory extension into the neck

**DOI:** 10.1186/s40792-024-01946-2

**Published:** 2024-11-05

**Authors:** Tomoki Keiya, Hirofumi Uehara, Miho Aoyagi, Atsushi Watanabe

**Affiliations:** 1 Department of Thoracic Surgery, Hakodate Goryoukaku Hospital, 38-3 Goryokakucho, Hakodate, Hokkaido 040-0001 Japan; 2 Department of Thoracic Surgery, Sapporo Medical University School of Medicine and Hospital, South 1, West 16, Chuo-Ku, Sapporo, Hokkaido Japan

**Keywords:** Cervicothoracic mature teratomas, Anterior mediastinal, Surgery

## Abstract

**Background:**

We present the case of a rare occurrence of an anterior mediastinal mature teratoma extending into the neck, commonly referred to as a cervicothoracic mature teratoma.

**Case presentation:**

A 19-year-old female presented with right-sided neck pain and swelling, which were found to be attributed to a 14 cm cystic lesion originating from the right thyroid lobe and extending into the mediastinum. A diagnosis of mediastinal teratoma with extension to the neck was made. Robot-assisted thymectomy was initiated but was complicated by dense tumor adherence to the superior vena cava and brachiocephalic veins, prompting a switch to a midline sternotomy. Simultaneous resection of the right thyroid lobe was performed due to inflammation. The transition to a midline sternotomy allowed successful excision of the tumor, which was confirmed to be a mature teratoma confined to the thoracic region. The patient's favorable postoperative course led to discharge on day 5 with no recurrence at nine months.

**Conclusions:**

Emphasizing the challenges and the importance of prompt intervention in the management of mediastinal teratomas with neck extension.

## Introduction

Mediastinal teratomas, commonly located in the superior or anterior mediastinum, are thought to arise from aberrant tissue in the thymus germ during embryonic development [1–3]. We report a case of a mature teratoma found in a cervicothoracic tumor of a young woman. However, mature cervicothoracic teratomas extending into the neck are rare. The etiology and extension patterns for these tumors remain unclear, with no established consensus to date.

## Case report

**Fig. 1:**
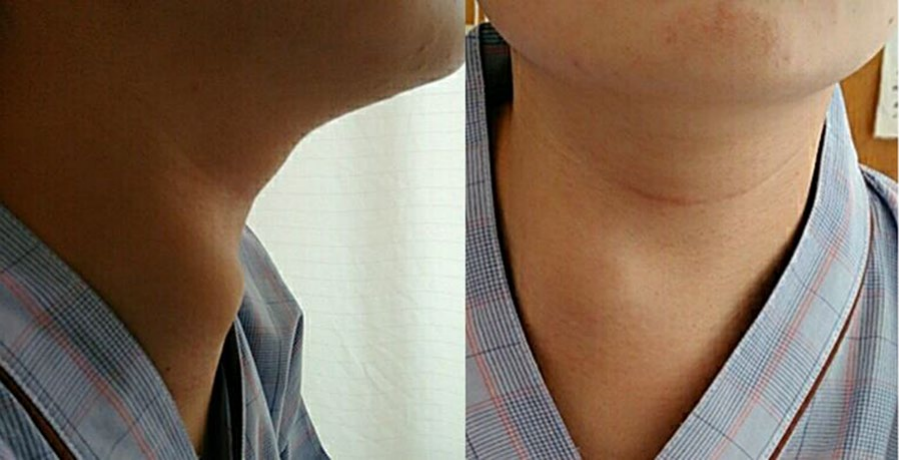
Preoperative physical examination revealed a tight and elastic swelling in the right cervical region

**Fig. 2:**
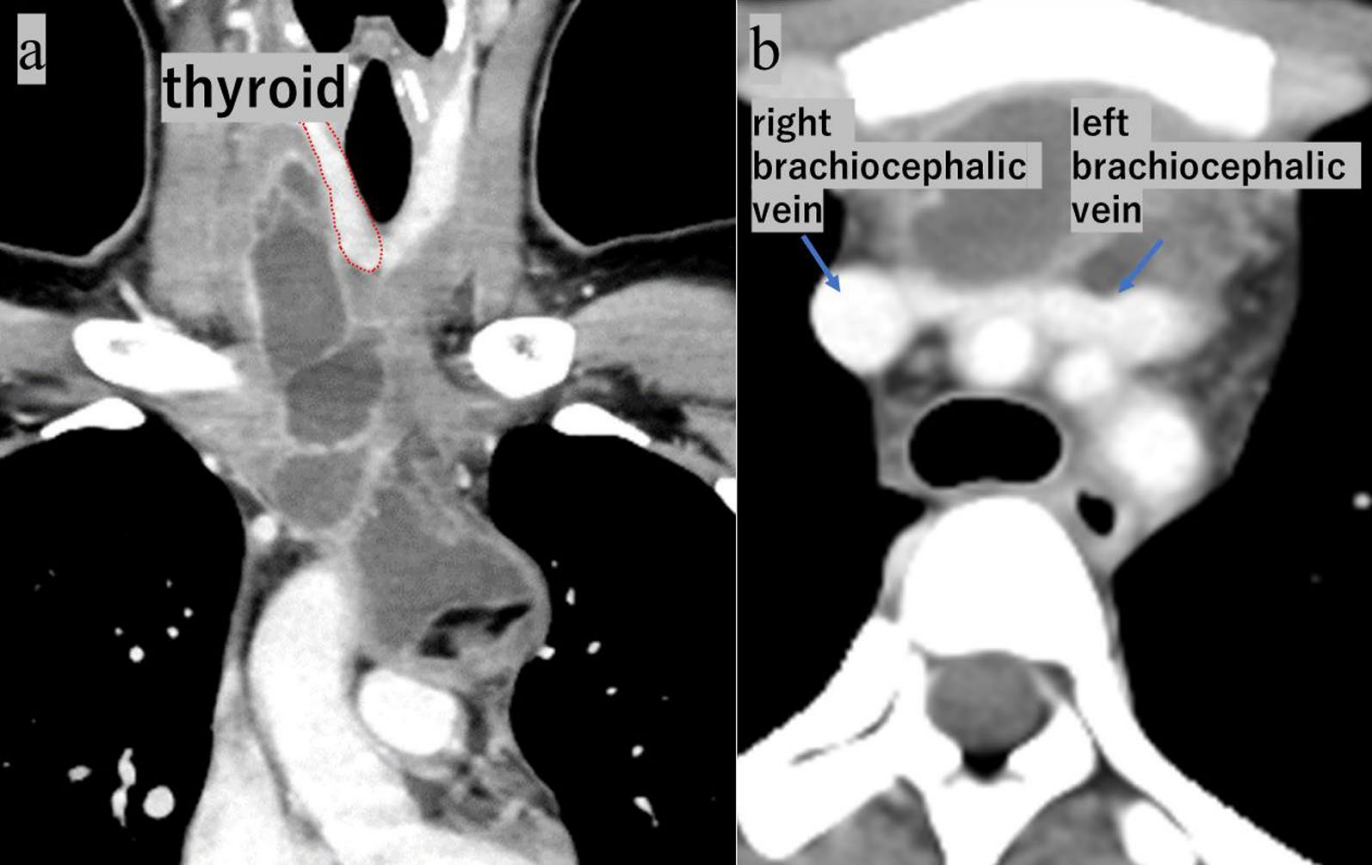
Contrast-enhanced neck and chest computed tomography revealed a multilocular cystic lesion. The lesion has extended to the right lobe of the thyroid (**a**) and is adjacent to both brachiocephalic veins (**b**)

**Fig. 3:**
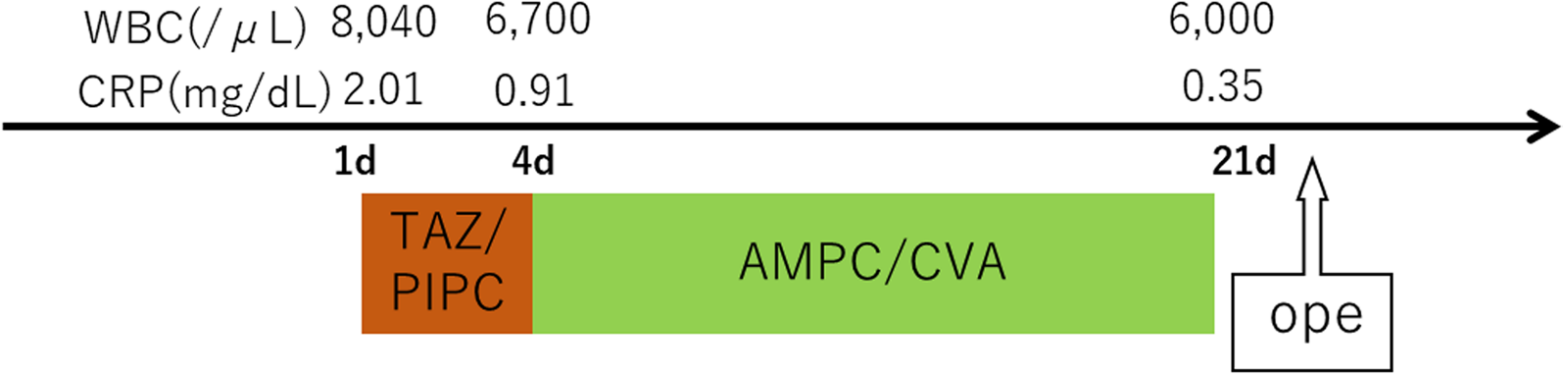
Inflammation and course of treatment from the date of admission to surgery. TAZ/PIPC, tazobactam/piperacillin; AMPC/CVA, amoxicillin/clavulanic acid

A 19-year-old female presented with chief complaints of right-sided neck pain and swelling, which led to consultation with a local physician (Fig. 1). Elevated inflammatory markers and the presence of a mass extending from the right neck to the mediastinum were noted, prompting referral to our institution. A contrast-enhanced CT of the neck and chest revealed a complex cystic lesion with a thick septum measuring 14 cm in cranio-caudal length. The lesion originated from the lateral aspect of the right lobe of the thyroid, deviated the trachea to the left, descended through the superior vena cava, brachiocephalic vein, and right brachiocephalic vein, crossed the midline to the left hemithorax, and extended to the pericardial reflection at the aortic arch (Fig. 2). Contrast MRI showed a homogeneous fluid component in the cephalic region, whereas the caudal region showed a heterogeneous mixture of fluid, soft tissue, and fat. Based on the imaging findings, a diagnosis of mediastinal teratoma with extension into the neck was made. Initial treatment included antibiotic therapy, which resulted in a reduction in the inflammatory response (Fig. 3). However, on day 21 after the initial presentation, the patient presented with a recurrence of neck swelling, necessitating urgent surgical intervention.

**Fig. 4:**
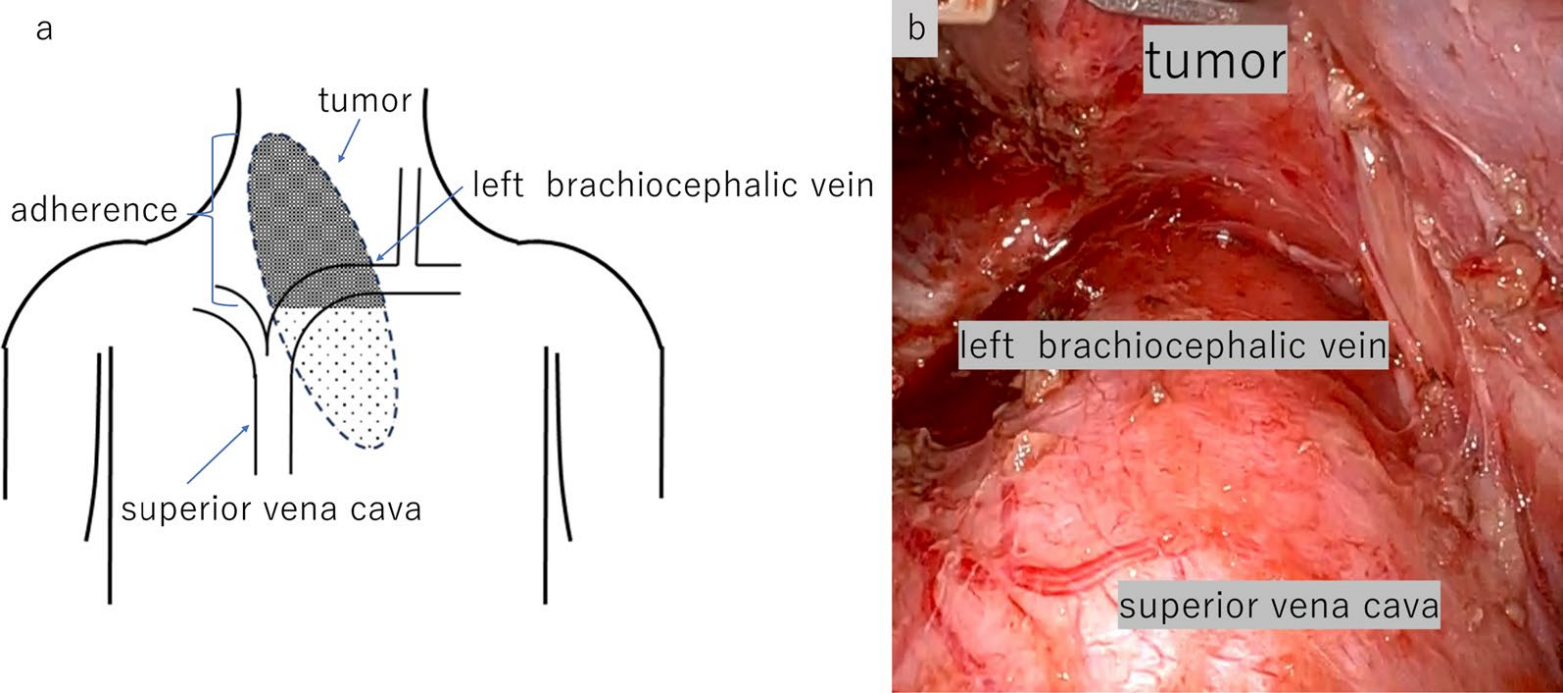
Schema (**a**) showing the relationship between the tumor and the left brachiocephalic vein. The tumor and left brachiocephalic vein were adherent and further dissection was difficult (**b**)

**Fig. 5:**
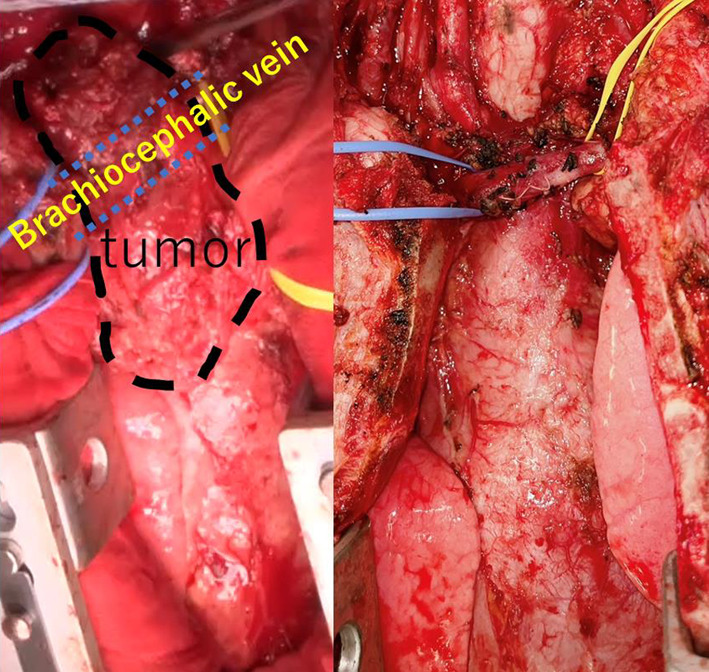
Intraoperative images. Transitioning to a midline sternotomy, with taping of the brachiocephalic vein. Successful and safe excision of the tumor

A robotic thymectomy was performed via a substernal approach. Dissection was achieved from the caudal aspect of the thymus, allowing for separation from the pericardium. However, the tumor was densely adherent to the superior vena cava and brachiocephalic veins. We added a cervical incision to secure the visualization of the cephalic aspect of the brachiocephalic vein. The dissection proceeded despite challenges posed by intense inflammation, which required concurrent resection of the right lobe of the thyroid. Attempts to perform robotic intrathoracic maneuvers were hampered due to difficulty visualizing the cephalic aspect of the brachiocephalic vein (Fig. 4). By switching to a midline sternotomy, the brachiocephalic vein was secured and preserved, and the tumor was successfully excised (Fig. 5). The operation was completed in 677 min with 465 ml of blood loss. Intraoperatively, adhesions with surrounding tissues were observed, and delaying surgery could have posed a risk of rupture. Therefore, prompt intervention is particularly crucial in cases of mediastinal teratomas extending into the neck.

The histopathological diagnosis was a mature teratoma, and the tumor portion within the thoracic cavity showed a variety of mature tissues, including adipose tissue, skin, respiratory epithelium, gastrointestinal mucosa, pancreatic tissue, bone, cartilage, and central nervous tissue. However, no abnormal tissue components were observed in the cystic wall of the neck.

The patient had a favorable postoperative course and was discharged on postoperative Day 5. Approximately 9 months postoperatively, the patient remains under outpatient observation with no evidence of recurrence.

## Comment

Mature teratomas occur in various sites and organs, including the ovaries, testes, retroperitoneal region, entire mediastinum, anterior sacral region, and coccygeal region [3].

In addition to this case, five other cases of mature teratomas extending from the anterior mediastinum to the neck have been reported [4–8] (Table 1). The age range is 19–29 years, with an average age of 23 years and the gender ratio was equal with 2 male and 3 female participants.

**Table 1:** Cases of cervicothoracic mature teratoma extending from the anterior mediastinum to the neck

Case	Author	Years	Age	Sex	Symptoms	Size (cm)	Approach
1	Hazama.K [4]	2002	27	Female	Neck swelling	Unknown	Cervical incision
2	Yamaguchi.K [5]	2002	28	Female	Neck swelling	Unknown	Cervical sternotomy
3	Rahman.M.T [6]	2004	29	Female	Neck swelling	10	Cervical sternotomy
4	Agarwal.G [7]	2008	19	Male	Neck swelling	16	Cervical sternotomy
5	Deng.H [8]	2021	21	Male	Neck swelling	9.6	Cervical sternotomy
6	Our case	2023	19	Female	Neck swelling, neck pain	14	Cervical sternotomy

Surgical removal was challenging due to inflammatory adhesions, necessitating thyroid lobe resection in our case. Due to the lack of histopathological diagnosis and the determination that surgery conducted after the acute phase of inflammation had subsided would be safer than emergency surgery, we opted against performing an urgent operation. Ultimately, opting for a semi-urgent surgical intervention based on imaging findings may have inadvertently facilitated the progression of adhesions over time. These findings underscore the importance of clinical vigilance, as teratomas are often silent manifestations. Surgical complexity underscores the need for meticulous preoperative planning and adaptability to unexpected complications. This series provides valuable insights into managing mature teratomas in atypical locations and encourages further collaborative research for refined strategies. The surgical intervention was initially commenced employing a robot-assisted modality, with the intention of circumventing the necessity for a midline sternotomy, taking into consideration the patient's youthful age and gender. Regrettably, this strategy ultimately led to the need for median sternotomy, resulting in an increase in surgical wound dimensions and prolonged procedural duration. We consider this to be a point for reflection in this case.

The extension pattern of mature teratomas from neck to anterior mediastinum entails two plausible scenarios. There is still no established consensus on the pattern of progression. Yamaguchi et al. proposed anatomically that the tumor advances into the neck because it is enclosed anteriorly by the sternum, posteriorly by the heart, left side by the aorta, with the solid part of the tumor located on the right side of the mediastinum, causing thickening of the right-side wall. While literature reports cases of teratomas extending from neck to anterior mediastinum [9], our case presents a unique pathology. Pathological analysis revealed teratoma components exclusively in the anterior mediastinal tumor, with the neck lesion primarily displaying inflammatory changes. Thus, we propose an extension from anterior mediastinum to neck.

Furthermore, 40–60% of mediastinal teratomas include pancreatic tissue [10, 11], and it is believed that both Langerhans cells and exocrine glands in the pancreatic tissue contribute to cyst formation and inflammatory reactions [12, 13]. There is also a hypothesis that tumors rich in pancreatic tissue pathologically may extend along the cervical region [5]. In this case, pancreatic tissue was observed in the surgical specimen, supporting this hypothesis.

In this case, the patient initially presented with only neck swelling and right-sided headache, and an elevation in inflammatory markers raised suspicion of cyst infection. Therefore, antibiotic therapy was initiated, and a semi-urgent surgical approach was planned after observing a decrease in inflammatory markers. Although the inflammatory response improved, an increase in neck swelling was noted during the waiting period for surgery. Intraoperatively, adhesions with surrounding tissues were observed, and delaying surgery could have posed a risk of rupture. Therefore, prompt intervention is particularly crucial in cases of mediastinal teratomas extending into the neck.

In this case of a benign mediastinal teratoma, the surgical approach was initially aimed at a less invasive robotic resection in a young patient. However, inflammatory changes in the thoracic cavity necessitated a change to a median sternotomy. In retrospect, starting with a sternotomy may have reduced overall operative time, highlighting the dynamic nature of surgical decisions balancing invasiveness and pragmatism in response to intraoperative conditions.

In our case report, direct extension of the cervicothoracic teratoma into the neck caused obvious inflammatory changes in the thyroid, but other mechanisms than direct infiltration should be considered for thyroid inflammation. One possible mechanism is the release from the teratoma of inflammatory mediators and substances that can affect thyroid tissue even in the absence of direct physical infiltration. These mediators could induce a local inflammatory response in the thyroid gland leading to the observed changes. Furthermore, the proximity of the teratoma to the thyroid gland may trigger an inflammatory cascade even in the absence of direct infiltration. However, no literature could be found to support these considerations.

## Conclusion

We report a case of a mature cervicothoracic teratoma requiring combined resection of the right thyroid lobe due to inflammatory extension into the neck. Although the occurrence of mediastinal teratoma extending into the neck is rare, the potential for rupture and compression of thoracic structures underlines the need for prompt surgical intervention.

## Data Availability

Not applicable.

## Abbreviation

CT: Computed tomography

## References

[1] Lewis BD, et al. Benign teratomas of the mediastinum. J Thorac Cardiovasc Surg. 1983;86:727–31. 6632945

[2] Davis RD Jr, Oldham HN Jr, Sabiston DC Jr. Primary cysts and neoplasms of the mediastinum: recent changes in clinical presentation, methods of diagnosis, management, and results. Ann Thorac Surg. 1987;44:229–37. 10.1016/s0003-4975(10)62059-0. 2820323 10.1016/s0003-4975(10)62059-0

[3] Tapper D, Lack EE. Teratomas in infancy and childhood. A 54-year experience at the Children’s Hospital Medical Center. Ann Surg. 1983;198:398–410. 10.1097/00000658-198309000-00016. 6684416 10.1097/00000658-198309000-00016 PMC1353316

[4] Hazama K, Miyoshi S, Ohta M, Matsuda H. Matured mediastinal teratoma extending into the cervical neck of an adult. Interact Cardiovasc Thorac Surg. 2003;2:265–7. 10.1016/s1569-9293(03)00053-7. 17670043 10.1016/S1569-9293(03)00053-7

[5] Yamaguchi K, et al. A cyst of benign mediastinal teratoma demonstrating a peculiar development: report of a case. Surg Today. 2002;32:159–62. 10.1007/s005950200011. 11998946 10.1007/s005950200011

[6] Rahman MT, Jaafar H, Naik VR, Ghazali MZ, Hassan S. Mature cystic teratoma: unusual presentation as anterior neck swelling. Singapore Med J. 2004;45:130–1. 15029417

[7] Agarwal G, Kar DK. Teratoma of the anterior mediastinum presenting as a cystic neck mass: a case report. J Med Case Rep. 2008;2:23. 10.1186/1752-1947-2-23. 18221571 10.1186/1752-1947-2-23 PMC2259367

[8] Deng H, Wang Z, Yang Q, Ye J. Mature cervical teratoma extending into the anterior mediastinum of an adult. Ear Nose Throat J. 2020;100:698–701. 10.1177/0145561320925563. 32425061 10.1177/0145561320925563

[9] Riaz A, et al. A rare presentation of cephalad extension of an anterior mediastinal teratoma—a case report. JPMA J Pak Med Assoc. 2019;69:902–4. 31201402

[10] Schlumberger HG. Teratoma of the anterior mediastinum in the group of military age; a study of 16 cases, and a review of theories of genesis. Arch Pathol. 1946;41:398–444. 21028277

[11] Suda K, Mizuguchi K, Hebisawa A, Wakabayashi T, Saito S. Pancreatic tissue in teratoma. Arch Pathol Lab Med. 1984;108:835–7. 6206822

[12] Sommerlad BC, Cleland WP, Yong NK. Physiological activity in mediastinal teratomata. Thorax. 1975;30:510–5. 10.1136/thx.30.5.510. 1198389 10.1136/thx.30.5.510 PMC470317

[13] Southgate J, Slade PR. Teratodermoid cyst of the mediastinum with pancreatic enzyme secretion. Thorax. 1982;37:476–7. 10.1136/thx.37.6.476. 6182623 10.1136/thx.37.6.476 PMC459346

